# Lemierre’s syndrome in a child

**DOI:** 10.12669/pjms.38.ICON-2022.5773

**Published:** 2022-01

**Authors:** Fatima Hemani, Anjum Naveed, Shakil Akhtar, Saba Shahid

**Affiliations:** 1 Dr. Fatima Hemani, MBBS. Resident, Department of Pediatrics, Indus Hospital & Health Network, Karachi, Pakistan; 2 Dr. Anjum Naveed, FRCS. Senior Consultant, Department of ENT, Indus Hospital & Health Network, Karachi, Pakistan; 3 Dr. Shakil Akhtar, FCPS. Junior Consultant, Department of ENT, Indus Hospital & Health Network, Karachi, Pakistan; 4 Dr. Saba Shahid MBBS, FCPS, MHPE. Head of Department, Department of Pediatrics, Indus Hospital & Health Network, Karachi, Pakistan

**Keywords:** Lemierre’s syndrome, Fusobacterium Necrophorum, Septic thrombosis

## Abstract

Lemierre’s Syndrome (LS) is a rare syndrome most frequently due to an anaerobic organism, Fusobacterium Necrophorum. It is commonly a complication of an acute oropharyngeal infection, but there are exceptions to its presentations. In our case the cause of LS was otitis media caused by Streptococcus species. This is a rather unusual presentation of LS. LS is caused due to septic complications of oropharyngeal infections, which lead to thrombophlebitis of internal jugular vein leading to thrombosis formation. In this case report, we present a case of Lemierre’s syndrome in a seven-year-old male child. The patient presented with high grade fever spikes and earache, which were unresponsive to oral antibiotics. LS was diagnosed in this patient on the basis of clinical, microbiological and radiological findings. After the diagnosis, treatment involved using broad spectrum antibiotics and anticoagulants, followed by surgery. Though role of anticoagulants is controversial in LS, but there is no specific guideline contraindicating the use of anti-coagulants. In our case, timely diagnosis and management enabled us to discharge the patient without any symptoms.

## INTRODUCTION

Andre-Alfred Lemierre first reported a combination of clinical features in twenty young patients.^1^ The clinical features included peritonsillar or pharyngeal abscess secondary to an anaerobic infection which led to thrombosis in internal jugular vein and septicemia. These symptoms were later coined as “Lemierre’s Syndrome (LS)”. Since the first reported case in 1936, LS became very rare due to development of antibiotics, and was once even termed as the “Forgotten Disease”. However, since the last two decades an increasing number of cases have been reported.^2^ It is unclear if this rising trend in cases is due to advancement in diagnostic techniques, decreased number of tonsillectomies or increased antibiotic resistance to organisms.^3^,^4^

We present a case of a 7-year-old boy, admitted with complaints of ear discharge and persistent fever spikes, which was later confirmed as a case of Lemierre’s syndrome on the basis of positive culture and radiological findings.

## CASE REPORT

A 7-year-old boy, with no significant past medical history, was admitted in the pediatric unit of our hospital with complaints of fever and right earache since eight days, along with mucopurulent discharge from the same ear for the last one day. On examination, he was a febrile, malnourished child with palpable cervical lymph nodes on the right side. The largest lymph node measured about 1.5 cm, was tender, smooth but hard in consistency. Detailed right ear examination revealed conductive hearing loss with a congested and perforated tympanic membrane with mucopurulent discharge.

**Fig.1a and b:**
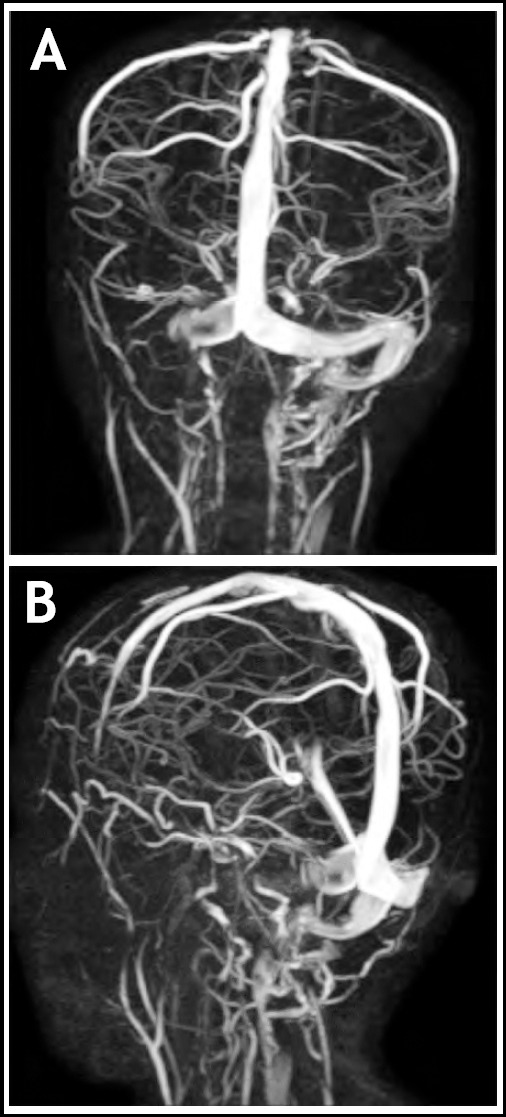
Posterior and lateral MRV images showing cerebral venous sinus thrombosis, involving right transverse sinus with extension into sigmoid sinus and internal jugular vein.

**Fig.2:**
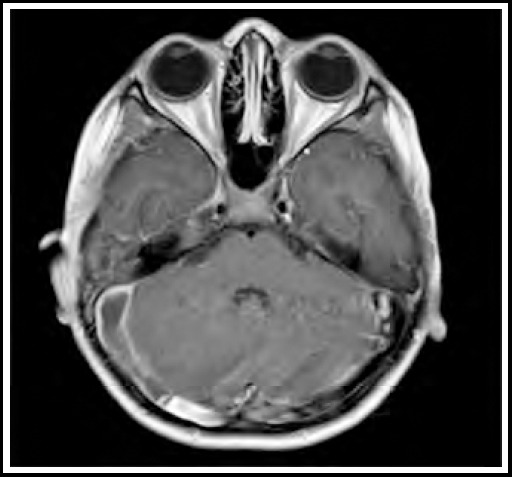
Post contrast enhancement in right cerebellar region.

On the basis of history and examination, our provisional diagnosis was otitis media. Intravenous injections of ceftriaxone and vancomycin were started, after which fever spikes spaced out, but persisted. Blood cultures which were sent on admission, came out positive for Streptococcus species which was sensitive to ceftriaxone. Culture of his ear discharge and repeated blood cultures were all negative, but due to persistent fever spikes, leukocytosis (TLC: 20.2 x 10^9^/L) and thrombocytopenia (105 x 10^9^/L). MRI and MRV showed cerebral venous sinus thrombosis, involving right transverse sinus with extension into sigmoid sinus and internal jugular vein (Fig.1a & 1b), along with focal right sided cerebellar meningeal and tentorial enhancement (Fig.2).

Explorative mastoidectomy revealed middle ear full of granulation tissue, extending to the mastoid antrum. Long process of incus was also eroded, while head of stapes was covered with granulation tissue. Lateral semicircular canal and facial canal were intact. Chest X-ray and echocardiography was done to screen for emboli in heart and pulmonary vasculature, which came out normal. The thrombus evident on MRI was treated with intravenous enoxaparin. After ten days of injection enoxaparin, our patient was switched to oral rivaroxaban to prepare him for discharge as his symptoms were starting to resolve. Later, he was discharged on oral antibiotics and was advised to continue anticoagulants for six more weeks. On follow up, his symptoms had completely resolved upon completion of treatment. We plan to follow him again after six months to plan for tonsillectomy.

## DISCUSSION

Lemierre’s Syndrome is also known as postanginal septicemia because sepsis usually manifests after an oropharyngeal infection.^5^ It usually begins as a local oropharyngeal infection, and presents with fever, dysphagia and neck swelling.^6^,^7^ Later during the course, infection spreads via blood stream to other organs. This spread of septic emboli usually presents with chest pain, hemoptysis and dyspnea in 79 to 100 percent cases,^7^ while less commonly deranged liver functions, arthralgia, and coagulopathies have also been reported.

The most commonly reported pathogen in LS is Fusobacterium spp., especially Fusobacterium Necrophorum.^7^ In a study published, of the 96 patients diagnosed with LS 52 percent were culture positive for Fusobacterium spp. The remaining cases is this study were positive for Streptococcus spp. (18%), and Staphylococcus aureus (6.3%), respectively.^7^ There are very few cases reported from Pakistan, especially in pediatric population.^8^ One of these reports, defined an unusual presentation of LS with Fusobacterium spp, but absence of jugular vein thrombosis.^6^ Another study was done in Abbottabad, where patients admitted with tonsillopharyngitis were screened for LS, and only two cases (1.28%) were identified. The first patient had throat swab culture positive for bacteroides and pseudomonas, while second patient’s culture showed no organism growth.^9^ In our case, LS was caused by Streptococcus species, which most likely was due to our patient’s unvaccinated status.

LS is diagnosed by a combination of clinical signs, positive blood cultures and radiological findings. Its treatment also involves broad spectrum antibiotics, surgical removal of septic focus and anticoagulants. Use of anticoagulants is still debatable, as no specific guidelines on treating LS are present.^10^,^11^ Many recent cases reported in pediatric population have used some form of anticoagulants,^12^-^14^ which seemed to have reduced or slowed the progression of thrombosis. The use of anticoagulants is still questionable though, as there is lack of evidence. Removal of thrombosis via surgery is hardly ever indicated, however surgery is indicated in removal of abscess in parapharygeal and tonsillar region, along with empyema of lungs or septic joints.^10^ These medical and surgical treatment options can be used separately or in combination to treat LS. In our case, due to persistent symptoms despite use of appropriate antibiotics, surgery was performed to remove infection. It is very important to treat LS at an early stage as delay in the treatment can be life threatening. The mortality rate of LS can vary from 5 to 18 percent.^7^,^10^

## CONCLUSION

LS is rather an unfamiliar syndrome with serious complications that can even cause death. It was once considered a rare syndrome, but due to increasing number of reported cases recently, it is important to spread awareness about this syndrome. It is vital to catch LS during its early stage to avoid any formidable complications. LS is usually reported after an oropharyngeal infection, but as seen in our case, it can occur as a sequelae to otitis media. It is therefore important to evaluate for LS in any patient presenting with a neck swelling or any other signs of inflammation after an oropharyngeal infection or otitis media.

### Authors’ Contribution:

**FH**: Conception of the idea, literature search, data compilation, write up and reviewing the article.

**AN, SH, SS:** Conception of the idea, review and final approval of the manuscript.

## References

[1] Zhao A, Samannodi M, Tahir M, Bensman S, Hocko M (2017). Lemierre's syndrome:Case report and brief literature review. IDCases.

[2] Karkos PD, Asrani S, Karkos CD, Leong SC, Theochari EG, Alexopoulou TD (2009). Lemierre's syndrome:A systematic review. Laryngoscope.

[3] Riordan T (2007). Human infection with Fusobacterium necrophorum (Necrobacillosis), with a focus on Lemierre's syndrome. Clin Microbiol Rev.

[4] Syed MI, Baring D, Addidle M, Murray C, Adams C (2007). Lemierre syndrome:two cases and a review. Laryngoscope.

[5] Rae J, Misselbrook K (2017). Lemierre's syndrome–a rare cause of disseminated sepsis requiring multi-organ support. J Intensive Care Soc.

[6] Rana MA, Kumar Y, Lashari AA, Mady AF (2017). Human infection with Fusobacterium necrophorum without jugular venous thrombosis:A varied presentation of Lemierre's syndrome. Case Rep Infect Dis.

[7] Johannesen KM, Bodtger U (2016). Lemierre's syndrome:Current perspectives on diagnosis and management. Infect Drug Resist.

[8] Farhan A, Shah YA, Ali BT, Mumtaz U, Farooq U (2016). The forgotten disease–Lemierre's syndrome. J Pak Med Assoc.

[9] Shah SA, Ghani R (2005). Lemierre's syndrome:A forgotten complication of oropharyngeal infection. J Ayub Med Coll Abbottabad.

[10] Allen BW, Anjum F, Bentley TP (2020). Lemierre syndrome. StatPearls [Internet].

[11] McGouran D, Keene A, Walklin R, Carter J (2013). A complex case of bilateral L emierre syndrome with suggestions on anticoagulation management. Intern Med J.

[12] Saurman VE, Kharayat P, Browning C, Butler A (2021). Not in Our Neck of the Woods:An Atypical Presentation of Lemierre's Syndrome. Am J Respir Crit Care Med.

[13] Hansberry DR, D'Angelo M, Prabhu AV, White MD, Tilwa S, Li Z (2020). Lemierre's syndrome:Acute oropharyngeal infection leading to septic thrombophlebitis of the internal jugular vein with pulmonary septic emboli. Interdiscip Neurosurg.

[14] Burdorf BT (2020). Lemierre's syndrome:A rare cause of septic emboli in a young adult. Radiol Case Rep.

